# Identification and Fine Mapping of Nuclear and Nucleolar Localization Signals within the Human Ribosomal Protein S17

**DOI:** 10.1371/journal.pone.0124396

**Published:** 2015-04-08

**Authors:** Scott P. Kenney, Xiang-Jin Meng

**Affiliations:** Center for Molecular Medicine and Infectious Diseases, Department of Biomedical Sciences and Pathobiology, College of Veterinary Medicine, Virginia Polytechnic Institute and State University (Virginia Tech), Blacksburg, Virginia, United States of America; University of North Texas Health Science Center, UNITED STATES

## Abstract

Human ribosomal protein S17 (RPS17) is mutated in Diamond-Blackfan Anemia (DBA), a bone marrow disorder that fails to produce sufficient red blood cells leading to anemia. Recently, an RPS17 protein sequence was also found to be naturally inserted in the genome of hepatitis E virus (HEV) from patients chronically-infected by HEV. The role of RPS17 in HEV replication and pathogenesis remains unknown due to the lack of knowledge about how RPS17 functions at a molecular level. Understanding the biological function of RPS17 is critical for elucidating its role in virus infection and DBA disease processes. In this study we probed the subcellular distribution of normal and mutant RPS17 proteins in a human liver cell line (Huh7). RPS17 was primarily detected within the nucleus, and more specifically within the nucleoli. Using a transient expression system in which RPS17 or truncations were expressed as fusions with enhanced yellow fluorescent protein (eYFP), we were able to identify and map, for the first time, two separate nuclear localization signals (NLSs), one to the first 13 amino acids of the amino-terminus of RPS17 and the other within amino acids 30-60. Additionally, we mapped amino acid sequences required for nucleolar accumulation of RPS17 to amino acids 60-70. Amino acids 60-70 possess a di-RG motif that may be necessary for nucleolar retention of RPS17. The results from this study enhance our knowledge of RSP17 and will facilitate future mechanistic studies about the roles of RSP17 in hepatitis E and DBA disease processes.

## Introduction

Ribosomes are ribonucleoprotein complexes responsible for protein translation. Cytoplasmic ribosomes are composed of a small 40S and large 60S subunit in eukaryotes comprising four RNA species and about 80 distinct proteins [1, 2]. Ribosome assembly is a complex process occurring in the nucleolus, nucleus, and cytoplasm of eukaryotic cells [3,4]. Genes encoding tRNAs are transcribed in the nucleoplasm by RNA polymerase III, undergo maturation, and are transported into the cytoplasm [5, 6]. Pre-mRNA genes, containing the sequences coding for the ribosomal proteins, are transcribed by RNA polymerase II in the nucleoplasm [7]. The pre-mRNAs are processed and transported as mRNPs to the cytoplasm where they are picked up by the ribosomes, and translated to produce ribosomal proteins. A subset of ribosomal proteins are then transported to the nucleolus [8]. The ribosomal RNAs (except 5S rRNA) are transcribed in the nucleolus as a precursor RNA by RNA polymerase I. The precursor is then processed to 18S, 5.8S and 28S rRNA. 5S rRNA is transcribed in the nucleoplasm by RNA polymerase III and transported to the nucleolus. The rRNAs are folded and associate with ribosomal proteins to form the 40S (including RPS17 and RPS19 proteins) and 60S ribosomal subunits. The subunits are then transported from the nucleolus to the cytoplasm (reviewed in [9]).

As ribosomal protein subunits possess an assembly step occurring in the nucleolus, it stands to reason that most, if not all ribosomal proteins, contain active nuclear import signals. Nuclear localization signals (NLSs) are amino acid sequences typically composed of positively charged lysines and/or arginines which allow proteins containing these signals to be actively trafficked from the cell cytoplasm to the nucleus [10]. There are two classes of NLSs, classical and non-classical. Classical NLSs bind to importin α which in turn binds to importin β allowing the ternary complex to interact with the nuclear pore complex for transport through the nuclear membrane [11]. Classical NLSs are further classified into monopartite and bipartite NLSs, monopartite defined as a singular stretch of charged amino acids typified by the consensus sequence K-K/R-X-KR [12]. Bipartite NLSs are typically two stretches of basic amino acids separated by a stretch of ten amino acids [13]. Non-classical NLSs do not require importin α for nuclear import instead relying on binding directly to importin β or another component of the nuclear import pathway [11,14].

Nuclear localization signals have been characterized within several ribosomal proteins. In humans, RPS6 [15], RPS7 [16], RPS19 [17], RPS25 [18], RPL5 [19], RPL7a [20], and RPL22 [21] have all been shown to possess nuclear localization signals. There is no clearly favored NLS for the trafficking of these ribosomal proteins. RPS25, RPL5, and RPL22 contain monopartite NLSs while RPS7 and RPS19 contain bipartite NLSs, RPS6 contains 2 monopartite, and one bipartite NLS. Currently, there is no information regarding whether RPS17 localizes to the nucleus/nucleolus at all and if so, what are the factors determining its subcellular localization.

Like the majority of ribosomal proteins, RPS17 is a basic protein of 135 amino acids and has a predicted molecular weight of 15.5 kDa. It is translated from five exons located on human chromosome 15 [22,23]. RPS17 is part of the 40S ribosomal subunit and lies in close proximity with RPS13, RPS16, and RPS19 which are involved in eIF-2 binding [24]. RPS17 along with RPS19 and RPS24 have been linked to diseases in humans including Diamond-Blackfan Anemia [25,26], an erythroid aplasia resulting in a deficiency of red blood cell precursors in the bone marrow. More recently, it has been found that a quasispecies of hepatitis E virus (HEV) genomes possess insertions of RPS17 and RPS19 sequences that bestow a growth advantage and expanded host tropism to these HEV strains *in vitro* [27] through an unknown mechanism.

To facilitate the study of the role of RPS17 in disease processes and virus infection in the future, here, in this study we have identified two distinct nuclear localization signals, a monopartite NLS within the first 13 amino acids and a complex bipartite NLS within amino acids 32 through 60. We further show that amino acids 60–70 contains a di-RG motif necessary for nucleolar localization of RPS17.

## Materials and Methods

### Expression vectors, plasmids, and cells

To assay active nuclear import, an expression vector generating three consecutive copies of eYFP, which produces a protein with a molecular weight of approximately 80 kDa (above the 50 kDa size limit for passive diffusion through the nuclear pore complex [28]) was created. The triple eYFP-N1 vector was created by amplifying eYFP from the eYFP-N1 vector (Clontech) using primers spk434 and spk436 (Table 1). The eYFP product was cloned into eYFP-N1 using *BsrGI* and *NotI* restriction sites. eYFP was subsequently amplified using primers spk484 and spk467 (Table 1) and inserted non-directionally into the double eYFP vector using the unique *NotI* restriction enzyme site resulting in three consecutive eYFP open reading frames (ORF). Human fibrillarin CFP [29] and human promyelocytic leukemia (PML) CFP were obtained from Dr. Leslie Parent (Pennsylvania State University College of Medicine, Hershey, PA) [30]. Huh7 human liver cells (passages 10–30) [31] were maintained in DMEM with 10% FBS and antibiotic/antimycotic.

**Table 1:** Oligonucleotide primer sequences used for PCR amplification and mutation of RPS17 and triple eYFP (restriction sites are underlined).

Primer ID	Primer Sequence (5’→3’)	Tm (°C)	Function
Spk383	CATCATCTCGAGATGGGCCGCGTTCG	64.9	RT-PCR RPS17
Spk384	CATCATCCGCGGCCAAACAGGTCCCCGAGG	70.1	RT-PCR RPS17
Spk407	CATCATAAGCTTTGCTATCTTGTTGCGGAGC	61.2	RPS17 39–51
Spk419	GACGCAAATGGGCGGTAG	56.7	eYFP OE PCR
Spk420	CCTCGCCGGACACGC	60.1	eYFP OE PCR
Spk421	CCACACGAACACACGCGTG	59.6	RPS17 KR32/33AA
Spk422	CACGCGTGTGTTCGTGTGG	59.6	RPS17 KR32/33AA
Spk425	CATCATCTCGAGATGAACGACTTCCACACGAACAAG	63.4	RPS17 39–51
Spk426	CATCATAAGCTTGTCGTTGCCCAGGCGC	65.2	RPS17 1–38
Spk427	CATCATCTCGAGATGCAGGTTACGTCACGCATCTG	64.9	RPS17 51–135
Spk434	CATCATTGTACACCATGGTGAGCAAGGGCGAG	65.0	eYFP (x2)
Spk436	CATCATGCGGCCGCCTCGTCCATGCCGAGAGT	71.6	eYFP (x2)
Spk467	GTATGGCTGATTATGATCTAGAGTCG	54.5	eYFP (x3)
Spk484	CATCATGCGGCCGCCGGAATGGTGAGCAAGG	70.3	eYFP (x3)
Spk505	CATCATAAGCTTCCGGGCCGCCTTCTTCAC	65.5	RPS17 1–13
Spk506	CATCATCTCGAGATGAGCAAAAAGCTCCGCAAC	63.5	RPS17 44–61
Spk507	CATCATAAGCTTAATTCGCTTCATCAGATGCG	59.4	RPS17 44–61
Spk512	CAAAACCGTGGCGGCGGCGGCC	70.9	RPS17 KK10/11AA
Spk513	GGCCGCCGCCGCCACGGTTTT	70.9	RPS17 KK10/11AA
Spk514	CCAGCGCAGCGCTCCGCAACAAG	67.7	RPS17 KK44/45AA
Spk515	CTTGTTGCGGAGCGCTGCGCTGG	67.7	RPS17 KK44/45AA
Spk516	CATCATCTCGAGATGGCGGCCCGGGTCATC	68.0	RPS 11–38
Spk517	CATCATTTCGAAGCTGGGGATAATGGCGATCTC	63.1	RPS 11–38

### Immunofluorescence staining of endogenous RPS17

Huh7 human liver cells were seeded into 6 well dishes with coverslips. One day later, the seeded cells were fixed in 4% paraformaldehyde for 15 minutes at 37°C followed by permeabilization with 0.25% Triton-X 100 at room temperature for 15 minutes. Cells were blocked in 5% nonfat dry milk for 15 minutes at 37°C, and subsequently probed using mouse monoclonal antibody raised against recombinant RPS17 of human origin sc-100835 (Santa Cruz Biotechnologies) at a 1:25 dilution at 37°C for 1 hour. Cells were washed once with PBS 0.1% tween 20, twice with PBS and probed with goat anti-mouse IgG (H+L) human serum adsorbed antibody conjugated to Alexa594 (Life Technologies) at a 1:250 dilution for 1 hour at 37°C. Cells were then incubated with 4', 6-diamidino-2-phenylindole (DAPI), washed thrice in PBS and mounted to slides using aqua polymount (Polysciences Inc).

### Construction of recombinant vectors expressing triple fluorescent-tagged RPS17 fusion proteins

Human *RPS17* was amplified from total RNAs extracted from the Huh7 liver cell line using a verso 1-step RT-PCR kit (Fisher Scientific) with primers spk383 and spk384 (Table 1). The resulting DNA fragment was cloned in frame using restriction sites *XhoI* and *HindIII* in the triple eYFP-N1 vector. Primers spk425 and spk407 were used to amplify RPS17 DNA coding for amino acids 39–51 and was inserted into the triple eYFP vector. Primer spk426 was used with primer spk383 to amplify *DNA coding for* amino acids 1–38 and primer spk427 was used with primer spk407 to amplify *DNA coding for* amino acids 51–135 for insertion into triple eYFP. Primers spk506 and spk507 (Table 1) were used to create RPS17 44–61. Primers spk516 and spk517 were used to create RPS17 11–38. gBlocks (Integrated DNA Technologies, Coralville, Iowa) with nucleotides coding for amino acids 1–13, 32–50, and 60–70 separated via a glycine, serine, glycine flexible linker were inserted into the triple eYFP vector using *Xho*I and *BamH*I restriction sites engineered at the 3’ and 5’ termini of the gBlocks. Construct 32–50 + 60–70 was also engineered with a methionine start codon to initiate translation.

### Mutagenesis of lysine and arginine residues within RPS17 triple eYFP fusion proteins

Overlap extension PCR using external primers within the eYFP vector spk419 and spk420 along with mutagenic primers within *RPS17* (spk421/422, spk512/513, spk514/515), were used to create mutants with lysines 10 and 11 to alanine, and lysines 44 and 45 to alanine. A gBlock gene fragment (Integrated DNA Technologies) containing sequences encoding full-length *RPS17* with the amino acid substitutions K/K 10/11, K/R 32/33, K/K 44/45, and K49 changed to alanine was cloned into the triple eYFP vector using *Xho*I and *BamH*I restrictions sites. Mutations R5A and K7A in RPS17 1–13 along with K59A/R60A in RPS17 amino acids 43–61 were created as gBlocks and subsequently inserted into the triple eYFP vector using *Xho*I and *Hind*III restriction sites.

### Transient transfection and microscopy

Huh7 human liver cells were seeded in 6 well dishes on coverslips. Cells were transiently transfected using either lipofectamine LTX (Invitrogen) or polyethylamine (Sigma Aldrich). At 24 hours post-transfection, cells were fixed in 4% paraformaldehyde for 15 minutes at 37°C, washed in phosphate buffered saline (PBS) once, stained with 20mg/ml DAPI (Sigma Aldrich) diluted 1:10,000 in PBS for 5 minutes at room temperature, washed twice more in PBS, and mounted on slides using aqua polymount (Polysciences Inc). Cells expressing DAPI and eYFP were imaged using a Nikon TE2000 microscope with a Prairie sweptfield confocal system and Elements software (Nikon, USA). Cells expressing eYFP and eCFP were imaged using a Zeiss LSM 510 confocal microscope with Zen 2009 software (Zeiss, USA).

## Results

### Prediction of nuclear localization signals within RPS17

**Fig 1:**
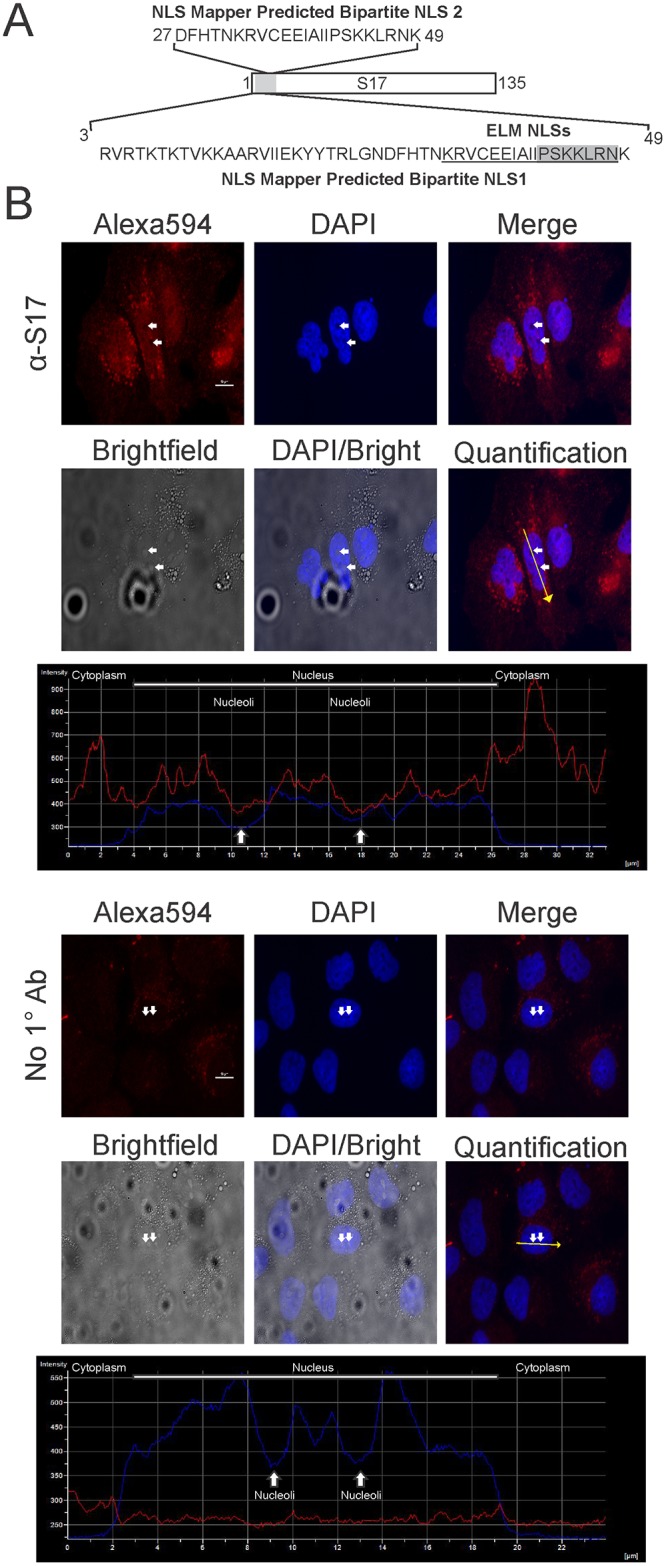
Schematic diagram and localization of human RPS17. (A) Schematic depiction of RPS17 with a blowout showing the amino acids implicated via NLS mapper software. Eukaryotic linear motif (ELM NLSs) predictions are underlined for the bipartite NLS and shaded for the monopartite NLS. (B) Immunofluorescent staining of Huh7 human liver cells with anti-RPS17 antibody (Top) or with no primary antibody (Bottom) observed with confocal microscopy. Fluorescent intensity in DAPI and Alex594 channels was quantified along a yellow line through one representative cell (Quantification) from both anti-RPS17 antibody or no primary antibody stained cells showing RPS17 signal (red line) with respect to the cytoplasm, nucleus, and nucleolus (blue line). RPS17 localized predominantly to punctate spots within the nucleus as evidenced by the ALEXA594 staining overlaid with DAPI and brightfield images. There was minor nonspecific staining of the anti-mouse Alexa594 antibody within the cytoplasm but none detected in the nucleus suggesting the staining was specific. Small white arrows correspond to the same pair of nucleoli in each panel and were used for the representative quantification. Scale bars on Alexa594 images represent 10 μm.

Two independent prediction methods were used to analyze the amino acid sequence from the human RPS17 protein. NLS mapper [32] predicted two bipartite NLSs: the first predicted motif occurred between amino acids 3 through 49 (probability score 5.5), and the second predicted motif occurred within amino acids 27 through 49 (probability score 6.1). Eukaryotic linear motif (ELM) analysis [33] also predicted two NLSs within the RPS17. The first ELM-predicted NLS is a bipartite NLS within amino acids 32 through 48 (KRVCEEIAIIPSKKLRN), and the second NLS is predicted to be a monopartite NLS encompassing amino acids 42 to 48 (PSKKLRN) (Fig 1A).

### Localization of endogenous RPS17 in Huh7 human liver cells

To demonstrate whether endogenous RPS17 protein localized to the nucleus within Huh7 human liver cells, we performed immunofluorescence staining on fixed cells probing with an antibody against the RPS17 protein. Cells probed with mouse anti-RPS17 followed by an anti-mouse FITC secondary antibody showed fluorescence within the cytoplasm, nucleus, and nucleolus (to a lesser extent) when imaged using confocal microscopy (Fig 1B). Cells that only received secondary antibody showed minor negligible background staining within the cytoplasm but none in the nucleus confirming that our staining was specific for RPS17 and that it is located within the nucleus. Because detection of S17 within the nucleus was difficult using immunostaining we switched to a system in which we could express S17 as a fusion with YFP to more clearly visualize subcellular localization.

### Experimental demonstration of nuclear import signals within the RPS17 protein and identification of subnuclear localization compartment

**Fig 2:**
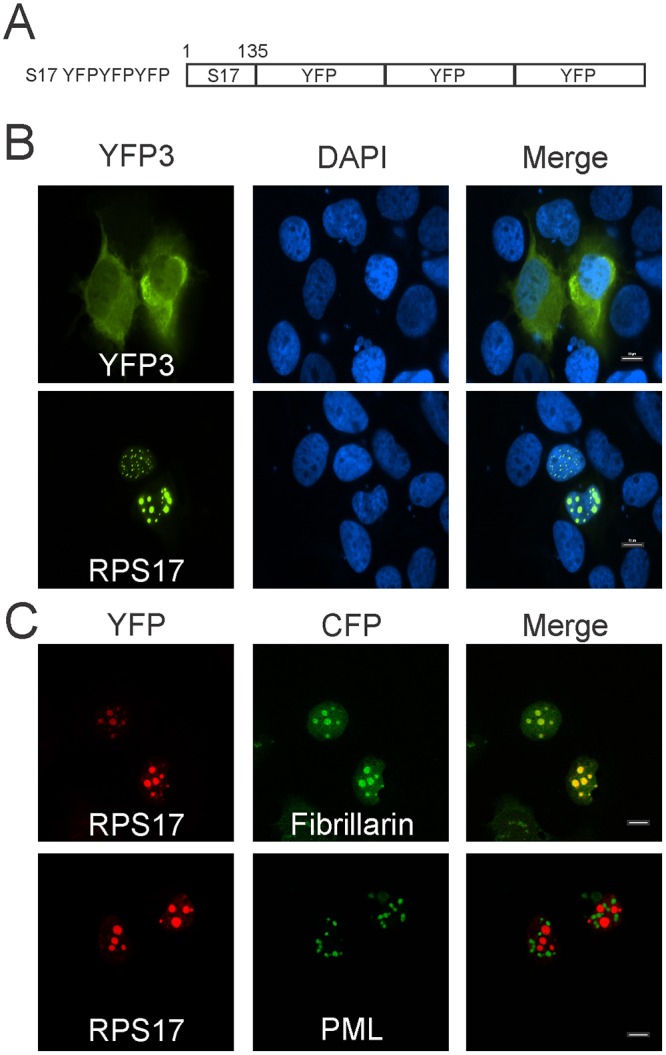
Subcellular localization of RPS17 attached to triple yellow fluorescent protein (eYFP). (A) Schematic depiction of RPS17 as a carboxy-terminal fusion to RPS17. (B) RPS17 was capable of trafficking triple YFP to punctate spots within the nucleus as observed via confocal microscopy. Triple eYFP alone was predominantly cytoplasmic (Top) whereas RPS17 triple eYFP was in punctate spots within the nucleus (Bottom). (C) RPS17 triple eYFP is located within nucleoli. RPS17 triple eYFP colocalized with fibrillarin CFP (nucleolar marker) but not promyelocytic leukemia (PML) protein (PML body marker). Scale bars on merged images represent 10 μm.

Based on our NLS predictions from NLS mapper and ELM analysis in conjunction with the detection of endogenous RPS17 expression in the nucleus, it appeared that RPS17 had an active nuclear localization signal. To experimentally demonstrate the existence of active nuclear import signals, we cloned specific fragments of the *RPS17* coding sequence in frame with three consecutive copies of enhanced yellow fluorescent protein (eYFP)(Fig 2A). For triple eYFP protein to be found in the nucleus, the amino acids attached to it must contain an active nuclear localization signal. As expected, when transiently expressed in Huh7 liver cells, triple YFP with no additional amino acid sequences localized predominantly to the cytoplasm (Fig 2B top). Full-length RPS17 protein attached to triple eYFP localized to distinct regions within the nucleus both large puncta resembling nucleoli and many smaller puncta that only occasionally appeared to coincide with nucleoli (Fig 2B bottom, data not shown). Subnuclear RPS17 eYFP colocalized with fibrillarin CFP [34], a nucleolar marker, but not promyelocytic leukemia (PML) CFP, a PML body marker (Fig 2C). This data suggests that RPS17 localizes to nucleoli.

### Mutation of basic amino acids predicted to be involved in nuclear import of RPS17 within the full length protein

To map the NLS sequences required for nuclear import of RPS17, we performed mutagenesis on basic amino acid residues (lysine and arginine) within the regions indicated by eukaryotic linear motif and NLS mapper software (Fig 1A). Altering lysine 10 and 11 to alanine in the context of the full-length RPS17 had no observable change in the localization of RPS17, as it remained predominantly nuclear (data not shown). Similarly, mutation of lysine 32 to alanine, lysines 44 and 45 to alanine, or lysine 49 to threonine in the context of full length S17 had no effect on the nuclear localization of RPS17 within Huh7 cells likely due to redundancy of the NLSs (data not shown).

### Mapping the nuclear localization signals through truncation mutations

**Fig 3:**
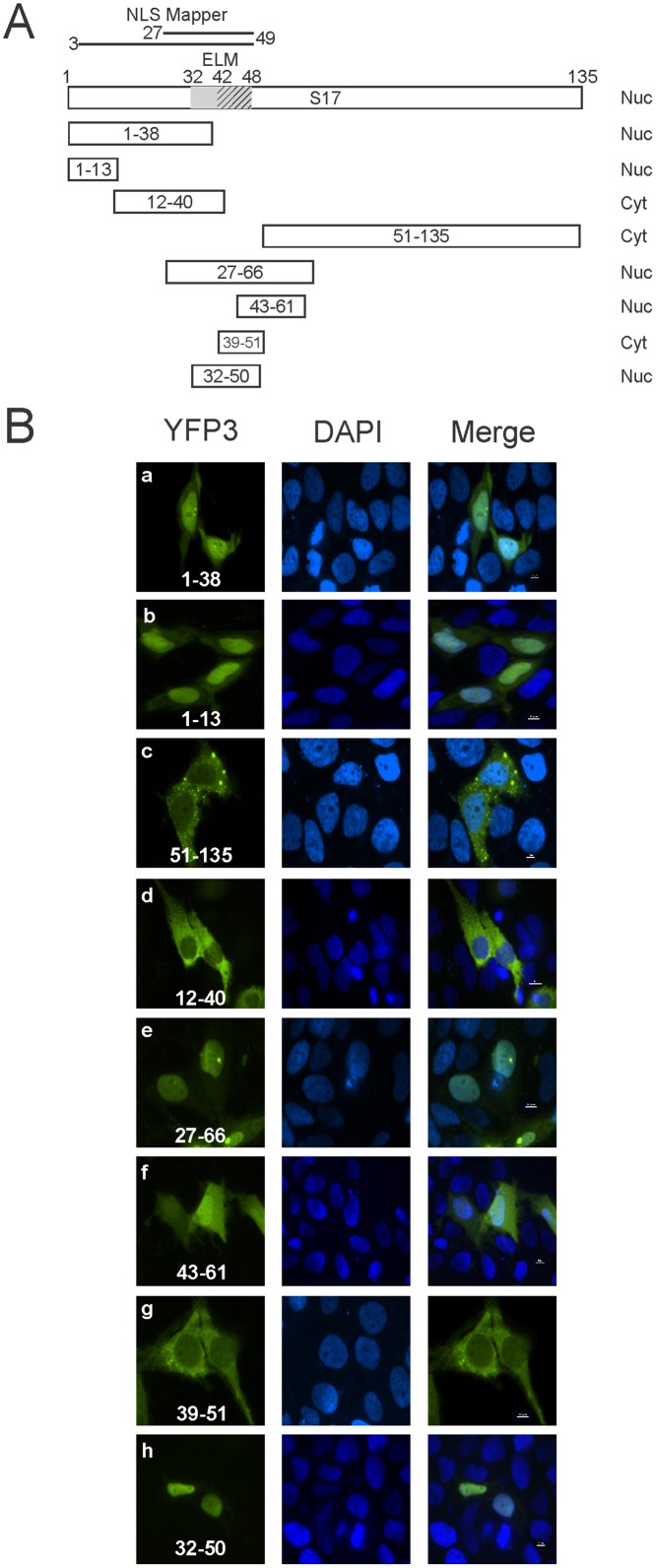
Trafficking of triple eYFP RPS17 protein truncations. (A) Schematic depicting RPS17 protein and tested truncations along with a summary of their cellular localization (Right). Nuc: nuclear; Cyt: cytoplasmic. NLS mapper predictions are represented by lines above the full-length protein, ELM NLS predictions are indicated by the shading within the full-length RPS17 representation. (B) Confocal microscopy images of cells expressing the indicated amino acids fused to triple eYFP listed in white. Amino acids 51–135, 12–40, 39–51 were not capable of actively trafficking triple eYFP to the nucleus whereas 1–13, 27–66, 43–61, and 32–50 were capable of trafficking triple eYFP into the nucleus. Scale bars on merged images represent 10 μm.

To define the specific amino acid sequences required for nuclear import, varying lengths of *RPS17* were cloned in frame with the triple eYFP protein allowing us to observe subcellular localization (Fig 3A). RPS17 amino acids 51 through 135 attached to triple eYFP remained cytoplasmic (Fig 3B panel c), suggesting that no nuclear trafficking motifs were present within the C-terminal 84 amino acids of RPS17. Amino acids 1–38 and 27–66 each resulted in nuclear localization of the triple eYFP protein, suggesting the existence of either an NLS within the overlapping amino acids 27–38 or multiple nuclear localization signals within the first 38 amino acids of RPS17 and within amino acids 27–66 (Fig 3B panels a and e respectively).

**Fig 4:**
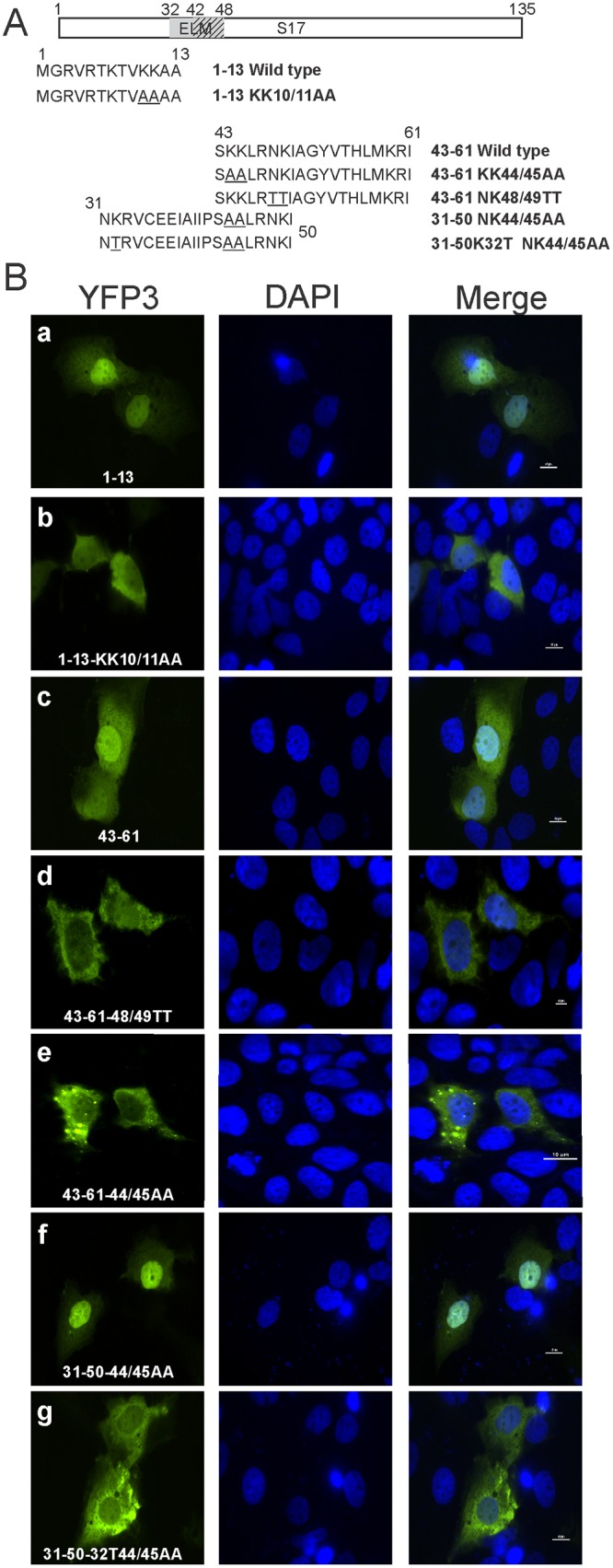
Localization of truncated RPS17 proteins containing amino acid mutations within putative nuclear localization signals. (A) Truncated RPS17 fragments that bestowed nuclear import to triple eYFP were mutated to determine the critical residues responsible for nuclear import. This schematic depicts the amino acid sequences of 1–13, 43–61, and 31–50 (capable of nuclear import) along with basic amino acids mutated to alanine or threonine. (B) Confocal microscopy images of RPS17 truncations with and without basic amino acids mutated to alanine or threonine. Lysines, 10/11, lysine and arginine 48/49, and lysines 44/45 were important for nuclear import in the context of amino acids 1–13, and 43–61 respectively, however, 44/45 was dispensable in the context of amino acids 32–50. Scale bars on merged images represent 10 μm.

To further map the nuclear localization signals within RPS17, we attached several smaller stretches of amino acids from RPS17 to eYFP3. To test whether the ELM predicted monopartite NLS (amino acids 42–48) (Fig 3A) was functional, we inserted *RPS17* encoding amino acids 39–51 in frame with *eYFP3*. RPS17 39–51 eYFP3 was localized to the cytoplasm suggesting that the predicted monopartite NLS was not sufficient for nuclear import (Fig 3 panel g). A slightly larger region of RPS17 encompassing amino acids 32–50 and 31–50 including the ELM predicted bipartite NLS (amino acids 32–48) was functional for nuclear import (Fig 3 panel h and data not shown). Surprisingly, another fragment encompassing amino acids 43–61 was also capable of importing triple eYFP into the nucleus, although it appears as though import or nuclear retention was less efficient as the protein was also diffuse within the cytoplasm (Fig 3B panel f and Fig 4B panel c).

To characterize the NLS within the first 38 amino acids, we added only the first 13 amino acids of RPS17 to eYFP. RPS17 1–13 triple eYFP maintained active nuclear import, suggesting that an NLS was within those 13 amino acid residues (Fig 3B panel b). Furthermore amino acids 12–40 were not capable of importing triple eYFP into the nucleus, suggesting that these amino acids did not possess an active NLS (Fig 3B panel d). These results suggested that RPS17 had at least 2 separate functional nuclear localization signals one within the first 13 amino acids and at least one complex bipartite NLS within amino acids 31–61.

### Fine mapping the amino acids required for nuclear import of RPS17

To map the NLS sequences required for nuclear import of RPS17, we performed mutagenesis on basic amino acid residues (lysine and arginine) located within the first 13 amino acids or within amino acids 31–51. Amino acids 1–13 attached to triple eYFP produced nuclear fluorescence (Fig 4B panel a), mutation of lysines 10 and 11 to alanine showed a localization that was predominantly cytoplasmic (Fig 4B panel b). Amino acids arginine 5 and lysine 7 mutations to alanine singly in the context of amino acids 1–13 remained diffusely nuclear, thus were not critical for nuclear import (data not shown). RPS17 fragments 32–50 and 43–61 tagged with triple eYFP were both localized to the nucleus (Fig 3B panel h and Fig 4B panel c, respectively). Within the context of only amino acids 43–61, separate mutation of lysines 44 and 45 to alanine or asparagine 48 and lysine 49 to threonine (Fig 4A) led to changes in localization from nucleus to cytoplasm (Fig 4B panels d and e). Unexpectedly, mutating lysines 44 and 45 to alanine in the context of amino acids 31–50 did not disrupt nuclear localization of the protein (Fig 4B panel f). Additional mutation of lysine 32 to threonine in conjunction with lysines 44 and 45 to alanine was necessary to prevent nuclear localization of amino acids 31–50 (Fig 4B panel g). Additionally, mutation of amino acids lysine 59 and arginine 60 in tandem within the context of amino acids 43–61 had no effect on nuclear localization of the protein (**data not shown**).

### Disruption of nuclear import within the full-length RPS17 protein

**Fig 5:**
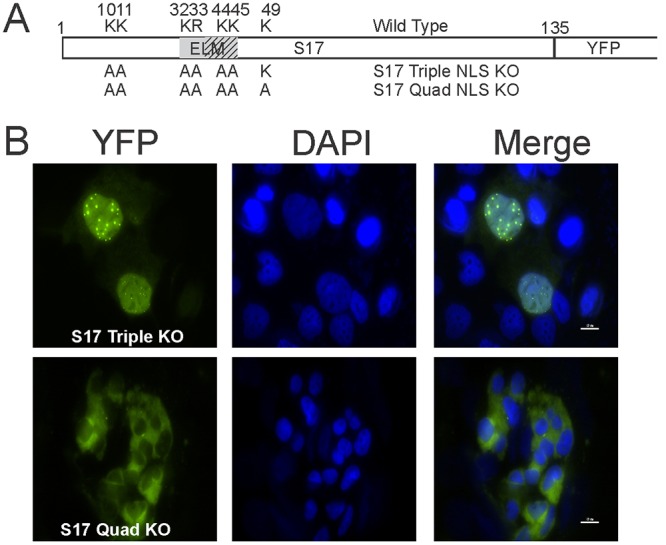
Localization of the full-length RPS17 containing point mutations fused N-terminally to single yellow fluorescent protein. (A) A schematic showing the amino acids found to be important for nuclear import in the context of the truncation mutants. Wild-type amino acids are shown above with amino acid number and residues altered to alanine are depicted below. (B) Confocal microscopy images of full-length RPS17 containing the denoted mutations fused with triple eYFP. Scale bars on merged images represent 10 μm.

After identifying the amino acid residues required for nuclear import within the context of RPS17 truncations, we next wanted to determine if these residues were also critical for nuclear import or if other residues played a role in the context of the full-length RPS17 protein. We first mutated lysines 10, 11, 32, 33, 44, and 45 to alanine (Fig 5A) and we could still detect eYFP fluorescence within the nucleus (Fig 5B top). We next mutated lysine 49 to alanine in addition to amino acids 10, 11, 32, 33, 44, and 45 to alanine. This RPS17 mutant was no longer capable of transiting into the nucleus (Fig 5B bottom).

### Determining the RPS17 sequence required for nucleolar localization

**Fig 6:**
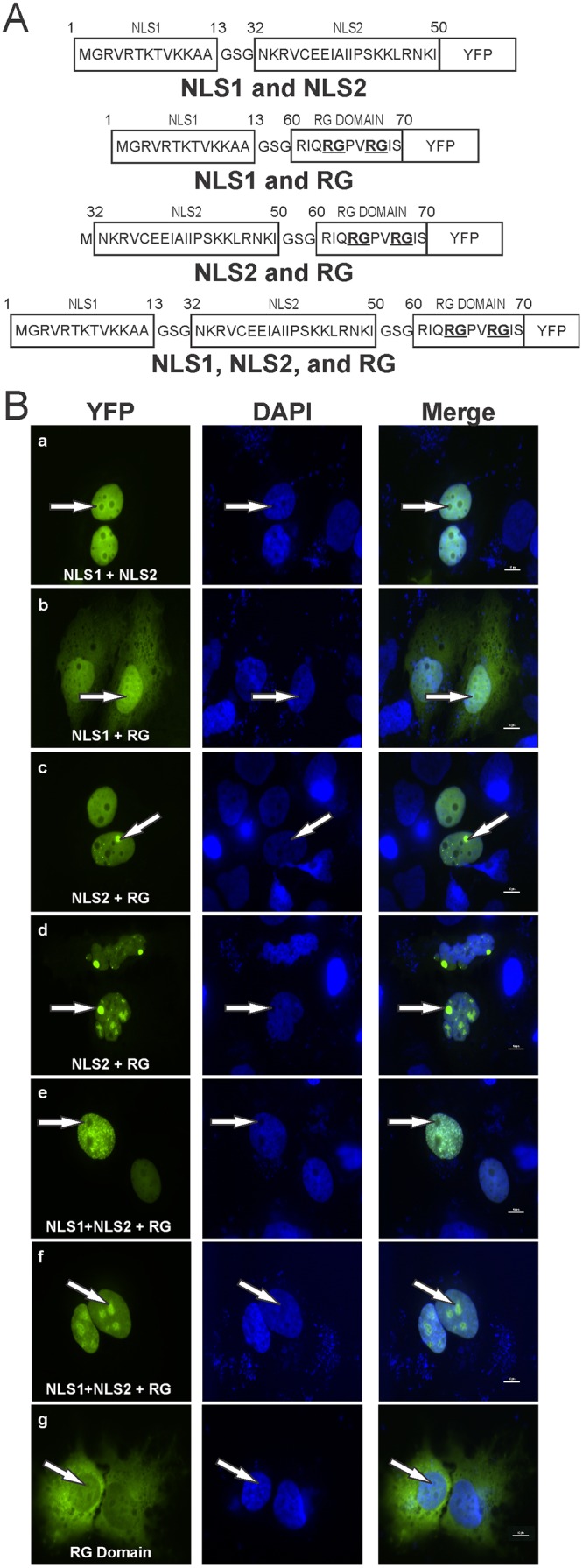
Mapping the determinants of nucleolar localization within RPS17. (A) Schematic depicting amino acids from nuclear localization sequence (NLS) 1 (amino acids 1–13) and NLS 2 (amino acids 32–50) in concert with each other separated via a glycine serine flexible linker or individually in the presence of the di-RG motif (amino acids 50–70). (B) Confocal microscopy images depicting localization of specified RPS17 amino acids attached to triple eYFP protein. Arrows denote the same single nucleoli in each set of panels. Scale bars on merged images represent 10 μm.

Truncation mutations that bestowed nuclear import functionality to triple eYFP produced a diffuse nuclear fluorescence (Fig 3B panels b, f, and h) and diffuse nuclear fluorescence with a reduced quantity of nucleolar localization (Fig 3B panel e) while the full-length RPS17 protein was strongly nucleolar during steady state conditions (Fig 2B bottom). This suggested that determinants other than single nuclear localization signals were required to retain the RPS17 protein within nucleoli. Therefore, we created constructs in which either amino acids 1–13 (NLS1) or amino acids 32–50 (NLS2) in the presence or absence of amino acids 60–70 (di-RG motif) to test whether two nuclear localization signals in tandem or if a single nuclear localization signal along with a di-RG motif [35] was sufficient for nucleolar trafficking (Fig 6A). Expressing amino acids 1–13 and 32–50 in tandem with triple eYFP resulted in diffuse nuclear fluorescence that excluded nucleoli (Fig 6B panel a). We further added amino acids 60–70, containing the di-RG motif, along with either NLS1 or NLS2. Interestingly, amino acids 1–13 in tandem with amino acids 60–70 resulted in both diffuse nuclear and nucleolar localization (Fig 6B panel b), whereas amino acids 32–50 in tandem with 60–70 produced diffuse nuclear localization with occasional nucleolar localization (Fig 6B panels c and d). Expressing amino acids 1–13, 32–50, and 60–70 in tandem resulted in different phenotypes including diffuse nuclear, punctate nuclear, and nucleolar staining (Fig 6B panels e and f). Expression of the RG domain alone remained predominantly cytoplasmic as expected since it did not contain a nuclear localization signal (Fig 6B panel g).

## Discussion

There are almost 700 proteins that have been implicated in ribosome biogenesis, nucleolar assembly, and regulation [15]. With so many proteins participating in the complex protein translational pathway, there are numerous areas requiring further elucidation. Here in this study we have shown that RPS17, a protein mutated in DBA and a host protein sequence hijacked by a unique strain of hepatitis E virus (HEV), contains at least two separate nuclear localization signals, one in the first 13 amino acids, and one or two within amino acids 32–60. Amino acids lysine 10 and 11 are critical for the function of the amino-terminal NLS whereas lysine 32, arginine 33, lysine 44, lysine 45, asparagine 48, and lysine 49 play roles in the NLS spanning amino acids 31–60. Amino acids arginine 5, lysine 7, lysine 59, and arginine 60 were not essential for nuclear import. Amino acids 60–70 containing a di-RG motif were necessary to localize RPS17 to nucleoli but not to recapitulate the strong nucleolar localization of the full-length RPS17 attached to triple eYFP.

The nuclear localization signal within the first 13 amino acids does not exactly match the K-K/R-X-KR motif typified by classical monopartite NLSs [14], although this is not surprising since many other classical NLSs do not match these consensus sequences either [36]. The KTV**KK** sequence of RPS17 more closely resembles a class 2 monopartite NLS [K(K/R)x(K/R)] [36], although even this consensus motif is not an exact match for the NLS within RPS17. Further research will be required to determine if this amino acid sequence does indeed bind with importin alpha or if RPS17 uses a different import mechanism for transport into the nucleus.

The nuclear localization signal between amino acids 31–50 (N**KR**VCEEIAIIPS**KK**LRNKI) completely fits with a bipartite NLS consensus sequence of KR (10-12X)K(K/R). However, it is surprising that we can alter amino acids lysine 44 and lysine 45 to alanine without disrupting NLS function. It is probable that the arginine and lysine residues at amino acids 46 and 48 may be aiding in creating the pocket which is binding to the importin and can withstand losing the lysines at amino acid 44 and 45.

Nucleolar trafficking is currently thought to be a two-step process with certain proteins. The first requirement is nuclear localization after which the protein interacts with a nucleolar protein and/or rRNA localizing the protein to the nucleolus [20, 21, 37–40]. Nucleolin, a non-ribosomal highly phosphorylated [41], and methylated [42] protein comprising as much as 10% of the nucleolar protein content [43], is thought to play a major role in the nucleolar import of ribosomal proteins, and has been shown to directly interact with some ribosomal proteins including L3 through L9, L13a, L18, L28, L35a, L37a, S3a, S8, S9, S11, and S26 [44]. No association between RPS17 and nucleolin was found by Bouvet et al in their study [44].

**Fig 7:**
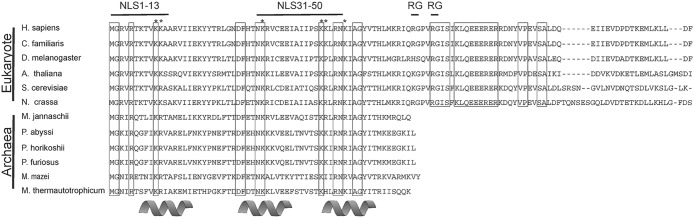
Alignment of RPS17 proteins from Eukaryotes and Archaea. Six RPS17 proteins from Eukaryotic organisms and six RPS17 proteins from Archae organisms were aligned using ClustalW. Organisms used were *Methanothermobacter thermautotrophicum* (O26894), *Methanococcus jannaschii* (P54026), *Pyrococcus abyssi* (Q9V0G0), *Pyrococcus horikoshii* (P58503), *Pyrococcus furiosus* (Q8U0U1), *Saccharaomyces cerevisiae* (P14127), *Neurospora crassa* (P27770), *Drosphila melanogaster* (P17704), *Arabidopsis thaliana RPS17C* (Q9SQZ1), *Canis Lupus familiaris* (P63273), and *Homo sapiens* (P08708). Absolutely conserved amino acids are boxed. Secondary structure of the protein is denoted below the alignment based on the solution structure of *M*. *thermoautotrophicum [47]*. Regions determined to be important for nuclear localization are shown above the alignments. Figure based on sequences originally analyzed by [47].

RPS17 contains a single di-RG motif within amino acids 60–70, and at least one RG motif was found in all of the eukaryotic RPS17 proteins we examined in this study (Fig 7). Di-RG motifs are quite common (found in >1700 proteins) and known to be involved in both protein-protein and protein-RNA interactions [35]. A significant number of nucleolar localized proteins contain this di-RG motif including serine arginine-rich splicing factors (NCBI 15055543), splicing factor 3B subunit 4 (NCBI 5032069), nucleolar protein 9 (NCBI 28212272), nucleolar RNA helicases (NCBI 379317177), nucleolar and coiled-body phosphoprotein 1 (NCBI 148596949), among others, suggesting that the RPS17 protein could be using a conserved mechanism for nucleolar localization. It is interesting to note that, in species where the first RG motif in RPS17 is not absolutely conserved, a charged amino acid remains in the R position (histidine for *D*. *melanogaster* and lysine for *A*. *thaliana* and *S*. *cerevisiae*) and an amino acid with a small side chain remains in the G position (serine in *D*. *melanogaster*). This could be indicative of a mechanism requiring a charge based interaction with little steric hindrance from the neighboring amino acid side chains.

We have shown that amino acids 60–70 containing the di-RG motif is capable of nucleolar localization in the presence of the NLSs encompassing amino acids 1–13 (NLS1) and 32–50 (NLS2), although NLS 1 did not produce prominent punctate staining as did NLS2. There could be several explanations as to why this could occur. Amino acids 1–13 did not appear to be as efficient at localizing eYFP to the nucleus as evidenced by diffuse cytoplasmic staining along with nuclear staining (Fig 6B panel B) whereas amino acids 32–50 was more efficient and had little fluorescence within the cytoplasm (Fig 6B panel c and d). It may also be that amino acids 1–13 and 32–50 use different nuclear import factors that somehow alter the final destination of the protein once in the nucleus. Additionally, if the import proteins binding to amino acids 32–50 remain attached longer than those involved in amino acid 1–13 import, there may be some type of steric blockage preventing the protein(s) which typically bind to the di-RG motif from binding, thus decreasing its ability to be retained within the nucleolus. As we could not recapitulate the intense nucleolar localization found when expressing the full-length RPS17 protein attached to triple eYFP with NLS1, NLS2, and the RG domain, it stands to reason that there are additional factors not located within amino acids 1–13, 32–50, and 60–70 that participate in nucleolar retention.

Another interesting observation in the present study is that the residues involved in nuclear import are conserved not only among all RPS17 proteins from Eukaryotic organisms but also Archaean organisms which do not have a cellular nucleus (Fig 7). This finding suggests that conserved residues may play critical roles in the structure or function of the RPS17 protein other than solely nuclear import. These amino acids would likely not be conserved in organisms that do not require nuclear import of ribosomal proteins. Like RPS19, regions of basic residues within RPS17 may be required for incorporation into pre-ribosomes or interacting with pre-ribosomal RNA [45]. Such a theory would also fit the mechanism of ribosomal assembly as assembled ribosomes would no longer require a nuclear localization signal with ribosomal complexes functioning primarily in the cytosol or endoplasmic reticulum.

In 25% of the cases of DBA, the S19 protein is mutated while only 1.4% of DBA patients have a mutation in RPS24 and approximately the same incidence (2.8%) occurs for RPS17 in DBA cases [25, 46]. The reason for higher prevalence of S19 mutation in DBA is still unknown. A single amino acid disruption of the nucleolar localization signal within RPS19 has been shown in DBA patients [17]. The ability of single point mutations altering S19 nuclear and nucleolar localization allows for a higher probability that a point mutation can adversely affect S19 function. Unfortunately, to date, there has been no published instance of mutations within the RPS17 nuclear or nucleolar localization signals contributing to DBA, only a T to G mutation disrupting the translational start codon of RPS17 [25]. The functional redundancy of both the nuclear and nucleolar localization signals within RPS17 is likely a contributing factor to its lesser prevalence in cases of DBA, although further work on the functionality of RPS17 lacking either one NLS or one RG domain needs to be performed to confirm this hypothesis.
